# Organisational Climate, Role Stress, and Public Employees’ Job Satisfaction

**DOI:** 10.3390/ijerph16101792

**Published:** 2019-05-21

**Authors:** Vicente Pecino, Miguel A. Mañas, Pedro A. Díaz-Fúnez, José M. Aguilar-Parra, David Padilla-Góngora, Remedios López-Liria

**Affiliations:** 1 Head Manager of HRM Office & IPTORA Research Team, University of Almería, 04120 Almería, Spain; vpecino@ual.es; 2 Department of Psychology & IPTORA Research Team, University of Almería, 04120 Almería, Spain; marodrig@ual.es (M.A.M.); pfunez@ual.es (P.A.D.-F.); 3 Department of Psychology, University of Almería, 04120 Almería, Spain; dpadilla@ual.es; 4 Department of Nursing Science, Physiotherapy and Medicine, Hum-498 Research Team, Health Research Centre, University of Almería, 04120 Almería, Spain

**Keywords:** organisational climate, role stress, employee’ well-being, public administration

## Abstract

The Job Demands-Resources (JD-R) model is an integrative theoretical framework for monitoring workplaces with the aim to increase job engagement and prevent burnout. This framework is of great interest since the management of job resources and demands can negatively affect employees, especially in organisational contexts characterised by high job demands. This study uses the job demands-resources model to investigate the relationships between organisational climate, role stress, and employee well-being (burnout and job satisfaction) in public organisations. This is a descriptive, cross-sectional study. The research participants are 442 public employees. A structural equation model was developed (organisational climate, job satisfaction, burnout, role stress). These confirm that organisational climate is correlated with role stress (−0.594), job satisfaction (0.746), and burnout (−0.408), while role stress is correlated with burnout (0.953) and job satisfaction (−0.685). Finally, there is a correlation between burnout and job satisfaction that is negative and significant (−0.664). The study confirms that a positive organisational climate could lead to less stressed and burned-out workers and, at the same time, to more satisfied employees with improved well-being.

## 1. Introduction

Reforms in the public sector have been a constant of the last 20 years, and there has been a particular focus on developing human factors [1]. These changes have become more necessary with the recent financial crisis, which positioned human resources management as a key internal element towards which many of the administration’s new policies should be directed [2,3].

Human factors are the key to improving public services. Bakker [4] states that people who want to change the world for the better often pursue a professional career in public service and are sometimes characterised by a ‘general altruistic motivation to serve the interests of a community of people, a state, a nation or humankind’ [5]. However, these employees face environments characterised by changes in performance expectations, high work demands, a hierarchical structure and bureaucratisation of work processes [6]; these factors tend to lead to the significant deterioration of employee well-being [7].

The emergence in the late twentieth century of the so-called ‘Positive Psychology’, which focuses on the positive side of people [8], was crucial to organisational and occupational psychology. This new paradigm has encouraged human resource departments to focus on increasing opportunities, satisfaction, motivation, resources and flexibility to develop people and enhance their well-being [9]. In this sense, we want to analyse how the stressful and motivational characteristics of work environments influence the well-being of employees, and how to create healthy environments [10].

### 1.1. The Job Demands-Resources Model

The Job Demands-Resources (JD-R) [11] model is an integrative theoretical framework for monitoring workplaces with the aim to increase job engagement and prevent burnout [12]. This framework is of great interest to Positive Organisational Psychology (POP) since the management of job resources and demands can negatively affect employees, especially in organisational contexts characterised by high job demands [13]. Demerouti et al. [11] defined job demands as ‘those physical, social, or organisational aspects of the job that require sustained physical or mental effort and are therefore associated with certain physiological and psychological costs’ ([11], p. 501). During the past three decades, many studies have shown that job characteristics can have a profound impact on employee well-being (i.e., job satisfaction, burnout, job engagement) and that job demands such as high work pressure, emotional demands, and role stress may lead to satisfaction problems and impaired health [14].

The job resources were defined as ‘those physical, psychological, social, or organisational aspects of the job that are either/or functional in achieving work goals; reduce job demands and the associated physiological and psychological costs; stimulate personal growth and development’ ([11], p. 501). The job resources are means to the achievement or protection of other valued resources and may be located at the level of the organisation at large, the interpersonal and social relations (i.e., supervisor and co-worker support, team climate), the organisation of work, and at the level of the task [14].

The JD-R model assumes that factors associated with organisations affect employees in two ways: A process of health deterioration, and a motivational process. In the first process, job demands predict the occurrence of burnout, which is associated with negative effects for the organisation and its employees. The motivational process links the existence of labour resources with the emergence of employee engagement, which leads to positive results for both employees and organisations [15].

### 1.2. Organisational Climate and Role Stress

One of the main job resources is the organisational climate [15]. Organisational climate research is interested in understanding the ways in which workers in an organisation experience and feel the climate, and how it is related to well-being. Schneider, Ehrhart and Macey [16] defined climate as ‘the shared meaning organisational members attach to the events, policies, practices, and procedures they experience and the behaviours they see being rewarded, supported and expected’. Thus, organisational climate shows how employees’ shared perceptions are connected to their work environment.

Role stress is the most studied job demand involving a natural phenomenon in organisations. It can be defined as a set of expectations, duties and obligations, applied to employees, coming from those who can influence the employees and help to define their roles [17]. It negatively affects employee efficiency [18], becoming an element that decreases employees’ well-being [19], and has equally pernicious effects on organisational performance [20]. The theory of role stress examines how characteristics of roles (e.g., conflict, ambiguity, and overload) are perceived and experienced as stressors by role incumbents, leading to affective and physiological symptoms as well as coping responses [21].

In the study of the relationship between organisational climate and role stress (job resources and job demands, respectively), the buffering hypothesis prevails. It suggests that the impact of job demands on employee well-being is weaker when they have a high level of resources [10]. The relationship between organisational climate and role stress is not a new one and has been shown to play a role in employee behaviour. Bakker and Demerouti [22] showed that the direction of this relationship can be positive or negative, and that the difference lies in the level of job resources (social support, job control and feedback) [12]. In this line, Hemingway and Smith [23] explored how organisational climate is associated with increased stress levels and the occurrence of negative behaviours for organisations; also, Pecino [24], using a sample of public employees, showed how organisational climate is strongly and negatively related to role stress.

### 1.3. The Motivational Process: Organisational Climate and Job Satisfaction

Job satisfaction is certainly the most researched topic in the history of industrial and organisational psychology [25]. The most relevant definition of job satisfaction is that offered by Locke [26], who defined it as a ‘positive or pleasant emotional state resulting from the subjective perception of the person’s work experiences’. This topic is of great importance in many models that seek to improve employee well-being [27] and organisational efficiency [28]. Many studies have shown that this variable has positive effects on organisations and their employees [29].

The influence of job resources (organisational climate) on job satisfaction, as an indicator of employee well-being, occurs through a motivational process [30]. Job resources seem to play a motivating role, fostering the learning, growth, and development of employees. Numerous studies have confirmed that the more positively employees perceive the organisational climate, the more satisfied they are [31].

### 1.4. The Health Deterioration Process: Role Stress and Burnout

Burnout is defined as ‘a syndrome of emotional exhaustion, depersonalization, and reduced personal accomplishment that can occur among individuals who work with other people’ [32]. There is growing evidence of the negative effects of burnout on employees and their performance [33]. Among the consequences for employees are low job satisfaction and deterioration of their health [34].

The relationship between job demands and burnout, as an indicator of employee well-being, occurs through the health deterioration process [30] and follows the line of the compensatory control model [35]. A study carried out by Buunk et al. [36] showed that intense or prolonged exposure to role stress causes psychological problems (i.e., burnout), as well as physical problems. Recent studies have deepened our understanding of the relationship between role stress and burnout in public employees, finding significant and positive results [37].

### 1.5. Cross-Links between Processes and Consequences

The organisational climate can affect employees’ well-being, directly influencing burnout. Under this hypothesis, Winnubst [38] carried out a pioneering study and concluded that organisational climate is an important antecedent of burnout. Recent studies have shown that formal support groups create organisational climates that decrease stressors such as burnout [39].

Also, the study of the relationship between role stress and job satisfaction is of major importance. Recently, several studies have presented evidence of this relationship in different country, industry, and labour contexts [40,41].

The JD-R model also provides a relationship between the consequences of job demands and those of job resources. A recent longitudinal study by Figueiredo-Ferraz et al. [42], who focused on analysing the relationship between burnout and job satisfaction, found a significant two-way relationship between these variables.

Other findings explained that—relative to private sector employees—public servants became the most engaged by intrinsic factors including work-related resources [43]. Work engagement significantly mediated the relationship between job resources and personal resources of public employees. Public sector workers were more strongly motivated by the desire to work in a supportive working environment. Most observed differences were explained by differences in job content such as skill variety, feedback, or task identity, not by the sector itself [43].

The aim of this study is to analyse the relationship between organisational climate, role stress, burnout, and job satisfaction in public employees. For this purpose, our hypotheses are consistent with previous studies:

Organisational climate will show a significant and negative reciprocal relationship with role stress (hypothesis 1) and a positive reciprocal relationship with job satisfaction (hypothesis 2). Also, role stress will show a significant and positive reciprocal relationship with burnout (hypothesis 3). Furthermore, organisational climate will show a significant and negative reciprocal relationship with burnout (hypothesis 4) and role stress will show a significant and negative reciprocal relationship with job satisfaction (hypothesis 5). Finally, job satisfaction will have a reciprocal relationship, both significant and negative, with burnout (hypothesis 6).

## 2. Materials and Methods

### 2.1. Participants and Procedure

Data were collected through online questionnaires in this cross-sectional study. Most participants in the chosen institution completed the questionnaire, for a total of 442 out of 475 public employees. All participants gave written informed consent in accordance with the Declaration of Helsinki. The Ethical Review Committee at the University of Almería (Spain) approved the study (UALBIO2018/027).

In terms of gender, 50.7% of participants were women and 49.3% men. For age, 5.7% of participants were under 36 years old 57.7% were between 36 and 45; 31.2% of them were between 46 and 55; and 5.4% were more than 55 years of age. As for educational level, 11.5% had no formal schooling, primary or secondary education; 25.8% had studied at high school; 61.1% were graduates; and 1.6% held a doctoral degree. In terms of labour status, 96.6% were civil servants and 3.4% were ordinary employees. Their positions at work were as follows: 86.4% were middle managers and workers and 13.6% were head managers. As for work hours, 392 of the participants (88.7 %) worked a morning shift and 50 (11.3%) worked an afternoon shift.

### 2.2. Instruments

#### 2.2.1. Organisational Climate

A shortened version of the First Organisational Climate/Culture Unified Search 93 (FOCUS-93) questionnaire by Van Muijen et al. [44] was used in this study. It consisted of 12 items using a 7-point Likert scale (ranging from 1 = ‘strongly disagree’ to 7 = ‘strongly agree’). The dimensions contained in the instrument were support, goals, innovation, and rules.

#### 2.2.2. Job Satisfaction

This variable was measured using CSLPS-EAP/33 [45], which consists of 33 items that are answered using a Likert scale with seven responses ranging from 1 = ‘strongly dissatisfied’ to ‘7 = strongly satisfied’. The dimensions contained in this instrument are team, retribution, means and conditions, intrinsic, business, workload, autonomy, and objects.

#### 2.2.3. Burnout

This was measured using an adapted version of the Maslasch Burnout Inventory [46], translated by Peiró et al. [47], which consists of nine items answered on a Likert scale with seven responses ranging from 0 = ‘never’ to 6 = ‘every day’. The dimensions contained in this instrument are exhaustion, cynicism, and professional efficacy.

#### 2.2.4. Role Stress

This was measured using a questionnaire composed by Rizzo et al. [48] that was translated by Peiró et al. [49]. It consists of 17 items with a response scale featuring 5 alternatives, ranging from 1 = ‘strongly disagree’ to 5 = ‘strongly agree’. The dimensions evaluated by this instrument are role ambiguity, role conflict and role overload. 

### 2.3. Statistical Analysis

In order to verify the reliability of the dimensions, the internal consistency was assessed using Cronbach’s alpha coefficient [50] for each variable and dimension. For each of the four variables under study, a structural equation model was fitted to confirm the dimensions. Finally, to check the relations between the different variables, a structural equation model was developed.

Structural equation models were fitted using the diagonalised weighted least squares (DWLS) [51] or robust maximum likelihood (RML) method [52], depending on the construct to be analysed, since the multivariate normality of the data was not met for any of the dimensions or variables. The univariate normality was checked for each item and skewness and kurtosis measures were obtained. To test the multivariate normality, the Henze-Zirkler test [53] was used.

The DWLS method requires large data sets; specifically, the sample size should be larger than *p* > (*p* + 1)/2, where p is the number of parameters to be estimated by the structural equation. Therefore, dimensions where the number of items considered in the model was too low could not be fitted using this method. Thus, the method used for those dimensions was RML. Those dimensions were from two variables: Climate and job satisfaction [54].

The goodness of fit for the proposed models was measured using the Tucker-Lewis Index (TLI) and the Comparative Fit Index (CFI). For both, values greater than 0.9 indicate a good fit. Another measure used was the Root Mean Square Error of Approximation, (RMSEA) where values lower than 0.08 indicate a good model fit and the upper limit of the 90% confidence interval should be below the 0.1 cut-off value for a good model fit [55]. The Standardized Root Mean Square Residual (SRMR) is an absolute measure of fit defined as the normalisation of the difference between the observed and predicted correlations. An SRMR value of less than 0.08 is considered as an adequate cut-off point for the goodness of fit [56]. The models were implemented using R software (R Foundation for Statistical Computing, Vienna, Austria) and the Lavaan package [57].

## 3. Results

First, we performed a confirmatory analysis of the dimensions for each of the variables used in the study, and later we presented a structural equation model for testing the relations between variables.

### 3.1. Confirmatory Analysis of Climate

Cronbach’s alpha for climate was 0.95. The RMSEA and SRMR obtained in the confirmatory analysis for climate were 0.079 (90% CI [0.076, 0.081]) and 0.076, respectively, indicating an acceptable data fit.

The estimated correlation for the dimension ranged from 0.824 between rules and innovation to 0.916 between goal orientation and innovation, indicating strong relationships between them. For rule dimension, item 7 had the lowest impact. Regarding the innovation dimension, items 8 and 28 had the lowest effects in the construct of the dimension. For the support and goal orientation dimension, all items had similar impacts on the constructs.

For the job satisfaction variable, the value of Cronbach’s alpha was 0.91. The goodness of fit values of the model were as follows: RMSEA = 0.057 (90% CI [0.053, 0.06]) and SRMR = 0.048. In addition, the values for CFI and TLI were 0.913 and 0.913, respectively, showing a good fit.

The estimated correlations between dimensions ranged from 0.319, between objects and means and conditions, to 0.826 between equipment and workload. The associations between intrinsic, firm, workload, autonomy, and objects were stronger than those between team retribution and conditions. Regarding the salary, autonomy and object dimensions, all items had similar impacts on their constructs. Within the environment/conditions and workload dimensions, the lower parameter estimated was associated with items 15 and 27.

### 3.2. Confirmatory Analysis of Role Stress

For role stress, the value of Cronbach’s alpha was 0.79. The RMSEA value was 0.034 (90% CI [0.023, 0.044]) and the SRMR value was 0.055. The values of CFI and TLI were 0.99 and 0.988, respectively, showing a good fit for the model. The correlations between the dimensions of the construct were positive, with a value of 0.167 for the dimensions of ambiguity and overload, and 0.508 for overload and conflict. Within the ambiguity dimension, the lower parameter estimate was associated with item 1; this item had a weaker impact on the dimension than the rest of them. Regarding the conflict dimension, item 7 had the weakest effect in the construct of the dimension. For the overload dimension, all items had a similar impact.

### 3.3. Confirmatory Analysis of Burnout

For the burnout variable, Cronbach’s alpha was 0.80. The RMSEA was almost 0 (90% CI [0.000, 0.031]), and the SRMR value was 0.038. The CFI and TLI values were both 1, indicating a perfect fit. The correlations between dimensions were positive, with a value of 0.16 for exhaustion and lack of personal accomplishment, and 0.459 for depersonalisation and exhaustion. Within the depersonalisation and lack of personal accomplishment dimensions, the lowest parameter estimated was associated with items 3 and 9; those items had a weaker impact on their respective dimensions than the rest of the items. For the exhaustion dimension, all items had a similar impact.

### 3.4. Structural Model of Climate as a Determinant of Job Demands

**Figure 1:**
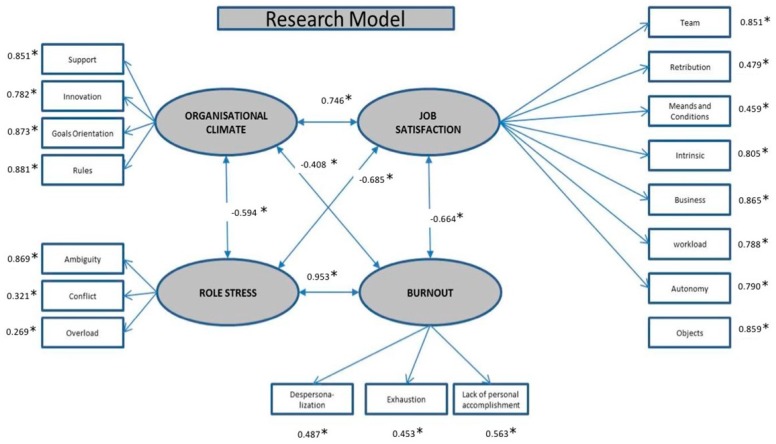
Standardised estimates and goodness of fit parameters for the Research Model. * *p*-value > 0.01. Crombach’s alpha = 0.766. Adjustment made with diagonalised weighted least squares (DWLS). Comparative fit index (CFI) = 0.967; Tucker-Lewis index (TLI) = 0.961; Root mean square error of approximation (RMSEA) = 0.064; Low 90 = 0.056, High = 0.072 and SRMR = 0.082.

The standardised estimates of the structural equation model testing the relations between all variables are presented in Figure 1. Cronbach’s alpha for the construct was 0.76. The measures of goodness of fit for the model were RMSEA = 0.064, (90% CI [0.056, 0.072]), SRMR = 0.082, CFI = 0.967 and TLI = 0.961. (Table 1). The climate variable had a significant direct negative effect on burnout and role stress, and a positive impact on satisfaction; it had a stronger impact on job satisfaction than on burnout and role stress. The correlation between burnout and role stress was positive, while the correlations between job satisfaction and burnout and role stress were negative.

## 4. Discussion

The aim of this work was to study the reciprocal relationships between a job resource (organisational climate), a job demand (role stress) and indicators of employee well-being (burnout and job satisfaction). The results confirmed that organisational climate is significantly and negatively linked to role stress (−0.594), which suggests that the existence of a positive climate would buffer role stress in workers.

In the JD-R model, the direct relationships between demands (role stress) and labour resources (organisational climate) are not specified as positive or negative correlations. Bakker and Demerouti [22] considered this as an empirical question related to the occupational context of the sample under study. Thus, in some organisational contexts, the relationship may be positive and in others negative, depending on variables such as the hierarchy, status, educational level and occupational sector. In the context of public employees, the results obtained confirmed the significant and negative relationships found by previous studies [58].

In this study, climate showed a significant and positive reciprocal influence on job satisfaction (0.746), confirming the motivational process of the JD-R model. Furthermore, previous studies showed that when employees perceive their organisational climate in a more positive way, they are more satisfied [31]. Also, we found that role stress and burnout are significantly and positively related (0.953). Thus, the higher the degree of role stress, the higher the incidence of burnout, which impairs employees’ health, as observed in the JD-R model and in previous studies [19,33].

The results confirmed that job demands and resources could interact to positively or negatively affect well-being in two cross-linked relationships [12]. On the one hand, organisational climate showed a significant and negative influence on burnout, which shows that a good climate is related to less burnout. Recent studies have shown that formal support groups create organisational climates that cushion employee stressors related to socio-emotional work such as burnout [59]. On the other hand, the results confirmed that role stress is significantly and negatively related to job satisfaction, which could indicate that higher role stress can lead to lower job satisfaction and, therefore, to worse employee well-being. This result is in line with the findings of recent studies [40,41] and clearly shows how reducing stress levels improves employee satisfaction.

Finally, the results confirmed that job satisfaction has a significant and negative relationship with burnout. In a recent longitudinal study, Figueiredo-Ferraz et al. [42] analysed the relationship between burnout and job satisfaction. They found a significant two-way relationship between the two variables, with more intense effects when job satisfaction was an antecedent of burnout rather than vice versa.

This study represents a step towards understanding the relationships between variables that affect the well-being of employees in public service. These results will be of great interest in the POP field as they will help to promote this viewpoint in the field of human resource management in public administration. The effects of job resources could enhance employees’ well-being, increasing satisfaction and preventing burnout [12].

### 4.1. Practical Implications

The results obtained in this study have important practical implications for managing the employees of public universities. A positive climate is key to enhancing and fostering the well-being of public employees, as the perception of a positive organisational climate leads to more satisfied employees. Such employees are less stressed and suffer from less burnout in their work, which should encourage those responsible for managing people to invest in creating comfortable and positive organisational climates. Such policies would not only help produce more positive results at work, but they would also enhance the development of work in a healthy environment. Furthermore, as was already mentioned when discussing the JD-R model, when a professional job is developed in a job environment characterised by the existence of role stressors, it will have negative effects on the health of employees. Therefore, it is important to establish policies in public administration that help employees have control over their demand level and increase their resources.

### 4.2. Limitations and Future Directions 

However, the results of our study should be considered with the following limitations in mind. First, the results were obtained from self-reports and might be affected by common method variance. Second, the sample was very specific and limited to public employees in a Spanish context; thus, the results cannot be generalised to other types of organisation or public administration. Third, the design of the study had a cross-sectional nature, which prevented us from drawing conclusions about the temporal order of effects and causality relationships. To avoid such limitations, future research should utilise a longitudinal methodology within a multi-method approach [34].

In our changing world, priority should be given to the study of how the well-being of employees in the public sector affects the execution of their duties. Also, the study of different mediating and moderating links in the relation between job demands, resources and well-being in the public context is important. Thus, a more inclusive approach should be taken to examining how work variables affect performance [60]. It will be interesting for future work to consider the multi-level relationships between attitudes, behaviours, and performance aggregated on the team level [61].

## 5. Conclusions

The study confirms that a positive organisational climate could lead to less stressed and burned-out workers and, at the same time, a higher role stress can lead to lower job satisfaction and, therefore, to worse employee well-being. Organisational climate showed a significant and positive influence on job satisfaction, confirming the motivational process of the JD-R model. 

## Figures and Tables

**Table 1:** Estimate parameters for the Job Demands-Resources Model.

Instruments	Variables	Estimated Loads	Std. Error	*Z*-Value	*p* (>|z|)	Standardised Estimates
Organisational Climate	Support	0.772	0.027	28.787	0.000	0.851
	Innovation	0.553	0.022	25.587	0.000	0.782
	Goal Orientations	0.733	0.026	28.741	0.000	0.873
	Rules	0.685	0.024	28.589	0.000	0.881
Job Satisfaction	Team	0.641	0.034	18.971	0.000	0.851
	Retribution	0.499	0.029	16.955	0.000	0.479
	Means and Conditions	0.450	0.028	16.152	0.000	0.459
	Intrinsic	0.611	0.033	18.468	0.000	0.805
	Business	0.688	0.036	19.247	0.000	0.865
	Workload	0.607	0.031	19.340	0.000	0.778
	Autonomy	0.609	0.033	18.680	0.000	0.790
	Objects	0.717	0.037	19.234	0.000	0.859
Role Stress	Ambiguity	0.511	0.063	8.081	0.000	0.869
	Conflict	0.238	0.030	7.934	0.000	0.321
	Overload	0.249	0.033	7.566	0.000	0.269
Burnout	Depersonalisation	0.413	0.039	10.574	0.000	0.487
	Exhaustion	0.436	0.041	10.535	0.000	0.453
	Lack of personal accomplishment	0.402	0.037	10.810	0.000	0.563
Burnout	Climate	−0.447	0.066	−6.782	0.000	−0.408
Job Satisfaction	Climate	1.121	0.079	14.227	0.000	0.746
Role Stress	Climate	−0.739	0.113	−6.568	0.000	−0.594

## References

[1] Truss C. (2008). Continuity and change: The role of the HR function in the modern public sector. Public Adm..

[2] Kickert W. (2012). State Responses to the Fiscal Crisis in Britain, Germany and the Netherlands. Public Manag. Rev..

[3] Lodge M., Hood C. (2012). Into an age of multiple austerities? Public management and public service bargains across OECD countries. Governance.

[4] Bakker A.B. (2015). A Job Demands-Resources Approach to Public Service Motivation. Public Adm. Rev..

[5] Rainey H.G., Steinbauer P. (1999). Galloping elephants: Developing elements of a theory of effective government organisations. J. Public Adm. Res. Theory.

[6] Caverley N. (2005). Civil service resiliency and coping. Int. J. Public Sect. Manag..

[7] Carlotto M.S., Gil-Monte P.R., Figueiredo-Ferraz H. (2015). Factor analysis of the Spanish Burnout Inventory among public administration employees. Jpn. Psychol. Res..

[8] Seligman M., Csikszentmihalyi M. (2000). Positive psychology: An introduction. Am. Psychol..

[9] Luthans F., Youssef C.M., Avolio B.J. (2007). Psychological Capital: Developing the Human Competitive Edge.

[10] Bakker A.B., Rodríguez-Muñoz A., Derks D. (2012). La emergencia de la psicología de la salud ocupacional positiva. Psicothema.

[11] Demerouti E., Bakker A.B., Nachreiner F., Schaufeli W.B. (2001). The Job Demands-Resources Model of Burnout. J. Appl. Psychol..

[12] Schaufeli W.B. (2017). Applying the Job Demands-Resources model: A ‘how to’ guide to measuring and tackling work engagement and burnout. Organ. Dyn..

[13] Bakker A.B., Hakanen J.J., Demerouti E., Xanthopoulou D. (2007). Job resources boost work engagement, particularly when job demands are high. J. Educ. Psychol..

[14] Bakker A.B., Demerouti E. (2007). The job demands-resources model: State of the art. J. Manag. Psychol..

[15] Bakker A.B., Demerouti E., Cooper C., Chen P. (2014). Job Demands-Resources Theory. Wellbeing: A Complete Reference Guide.

[16] Schneider B., Ehrhart M.G., Macey W.H., Zedek S. (2010). Perspectives on Organizational Climate and Culture. Handbook of Industrial and Organizational Psychology.

[17] Katz D., Kahn R.L. (1978). The Social Psychology of Organizations.

[18] Kahn R., Wolfe D., Quinn R., Snoek J., Rosenthal R. (1964). Organizational Stress: Studies on Role Conflict and Ambiguity.

[19] Díaz-Fúnez P.A., Pecino V., Mañas M.A. (2016). Ambigüedad de rol, satisfacción laboral y ciudadanía organizacional en el sector público: Un estudio de mediación multinivel. Rev. Psicología.

[20] Örtqvist D., Wincent J. (2006). Prominent consequences of role stress: A meta-analytic review. Int. J. Stress Manag..

[21] Bliese P.D., Edwards J.R., Sonnentag S. (2017). Stress and well-being at work: A century of empirical trends reflecting theoretical and societal influences. J. Appl. Psychol..

[22] Bakker A.B., Demerouti E. (2017). Job demands-Resources Theory: Taking Stock and Looking Forward. J. Occup. Health Psychol..

[23] Hemingway M.A., Smith C.S. (1999). Organizational climate and occupational stressors as predictors of withdrawal behaviours and injuries in nurses. J. Occup. Organ. Psychol..

[24] Pecino V. (2016). Clima Organizacional y sus Consecuencias: Estudios Multinivel en el Sector Público. Unpublished. Ph.D. Thesis.

[25] Judge T.A., Klinger R., Eid M., Larsen R.J. (2008). Job satisfaction: Subjective well-being at work. The Science of Subjective Well-Being.

[26] Locke E.A., Dunnette M.D. (1976). The Nature and Causes of Job Satisfaction. Handbook of Industrial and Organizational Psychology.

[27] Wright T.A. (2005). The role of “happiness” in organizational research: Past, present and future directions. Res. Occup. Stress Well-Being.

[28] Bowen D.E., Ostroff C. (2004). Understanding HRM-firm performance linkages: The role of the “strength” of the HRM system. Acad. Manag. Rev..

[29] Bowling N.A., Eschleman K.J., Wang Q. (2010). A meta-analytic examination of the relationship between job satisfaction and subjective well-being. J. Occup. Organ. Psychol..

[30] Schaufeli W.B., Bakker A.B. (2004). Job demands, job resources, and their relationship with burnout and engagement: A multi-sample study. J. Organ. Behav..

[31] Pecino V., Mañas M.A., Díaz-Fúnez P.A., López-Puga J., Llopis J.M. (2015). Clima y satisfacción laboral en el contexto universitario. An. Psicología.

[32] Maslach C., Leiter M.P. (2008). Early predictors of job burnout and engagement. J. Appl. Psychol..

[33] Gabris G.T., Ihrke D.M. (2001). Does performance appraisal contribute to heightened levels of employee burnout? The results of one study. Public Pers. Manag..

[34] Ganster D.C., Rosen C.C. (2013). Work stress and employee health: A multidisciplinary review. J. Manag..

[35] Hockey G.R.J. (1997). Compensatory control in the regulation of human performance under stress and high workload: A cognitive-energetical framework. Biol. Psychol..

[36] Buunk B.P., Jonge J., Ybema J.F., Wolf J., Drenth P.J.D., Thierry H. (1998). Psychosocial Aspects of Occupational Stress. Handbook of Organization and Work Psychology.

[37] Guthrie C.P., Jones A. (2012). Job Burnout in Public Accounting: Understanding Gender Differences. J. Manag. Issues.

[38] Winnubst J.A., Schaufeli W.B., Maslach C., Marek T. (1993). Organizational structure, social support, and burnout. Professional Burnout: Recent Developments in Theory and Research.

[39] Drach-Zahavy A. (2010). How does service workers behavior affect their health? Service climate as a moderator in the service behavior-health relationships. J. Occup. Health Psychol..

[40] Orgambídez-Ramos A., Borrego-Alés Y., Mendoza-Sierra I. (2014). Role stress and work engagement as antecedents of job satisfaction in Spanish workers. J. Ind. Eng. Manag..

[41] Singh A.P., Singhi N. (2015). Organizational role stress and social support as predictors of job satisfaction among managerial personnel. J. Psychosoc. Res..

[42] Figueiredo-Ferraz H., Grau-Alberola E., Gil-Monte P.R., García-Juesas J.A. (2012). Burnout and job satisfaction among nursing professionals. Psicothema.

[43] Buelens M., Broeck H.V. (2007). An analysis of differences in work motivation between public and private sector organizations. Public Adm. Rev..

[44] Van Muijen J., Koopman P., De Witte K., De Cock G., Susanj Z., Lemoine C., Turnipseed D. (1999). Organizational culture: The Focus Questionnaire. Eur. J. Work Organ. Psychol..

[45] Lloret S., González-Romá V., Peiró J.M. (1993). El cuestionario CSLPS-EAP/33: Un Estudio acerca de su validez. Psicológica.

[46] Maslach C., Jackson S.E. (1986). MBI: Maslach Burnout Inventory.

[47] Peiró J.M., González-Romá V., Tordera N., Mañas M.A. (2001). Does role stress predict burnout over time among health care professionals?. Psychol. Health.

[48] Rizzo J.R., House R.J., Lirtzman S.I. (1970). Role conflict and ambiguity in complex organizations. Adm. Sci. Q..

[49] Peiró J.M., Meliá L., Torres M.A., Zurriaga R. (1986). La medida de la experiencia de la ambigüedad en el desempeño: El cuestionario general de ambigüedad de rol en ambientes organizacionales. Evaluación Psicológica.

[50] Cronbach L.J. (1951). Coefficient alpha and the internal structure of the tests. Psychometrika.

[51] Jöreskog K.G., Sörbom D. (1996). LISREL 8 User’s Reference Guide.

[52] Maas C.J.M., Hox J.J. (2004). Robustness issues in multilevel regression analysis. Stat. Neerl..

[53] Henze N., Zirkler B. (1990). A class of invariant and consistent tests for multivariate normality. Commun. Stat. Theory Methods.

[54] Hox J.J., Maas C.J.M., Brinkhuis M.J.S. (2010). The effect of estimation method and sample size in multilevel structural equation modeling. Stat. Neerl..

[55] Hu L.T., Bentler P.M. (1999). Cutoff Criteria for Fit Indexes in Covariance Structure Analysis: Conventional Criteria Versus New Alternatives. Struct. Equ. Model..

[56] Hooper D., Coughlan J., Mullen M. (2008). Structural Equation Modelling: Guidelines for Determining Model Fit. Electron. J. Bus. Res. Methods.

[57] Rosseel Y. (2012). Lavaan: An R package for structural equation modeling. J. Stat. Softw..

[58] Pecino V., Díaz P.A., Mañas M.A. (2017). Clima, Estrés y Satisfacción Laboral: Un Estudio Multinivel en el Sector Público. Rev. Psicol. Soc..

[59] Drach-Zahavy A. (2008). Workplace health friendliness: A cross level model for predicting workers’ health. J. Occup. Health Psychol..

[60] Rosen C.C., Chang C.-H., Djurdjevic E., Eatough E. (2010). Occupational stressors and job performance: An updated review and recommendations. Res. Occup. Stress Well Being.

[61] Whitman D., Van Rooy D.L., Viswesvaran C. (2010). Satisfaction, Cirizenship Behaviors, and Performance in Work Units: A Meta-Analysis of Collective Construct Relations. J. Pers. Psychol..

